# Targeting Cell Survival Proteins for Cancer Cell Death

**DOI:** 10.3390/ph9010011

**Published:** 2016-02-25

**Authors:** Manoj K. Pandey, Sahdeo Prasad, Amit Kumar Tyagi, Lokesh Deb, Jiamin Huang, Deepkamal N. Karelia, Shantu G. Amin, Bharat B. Aggarwal

**Affiliations:** 1 Department of Pharmacology, College of Medicine, Pennsylvania State University, 500 University Drive, Hershey, PA 17033, USA; dkarelia@hmc.psu.edu (D.N.K.); sga3@psu.edu (S.G.A.); 2 Department of Experimental Therapeutics, Cytokine Research Laboratory, The University of Texas MD Anderson Cancer Center, Houston, TX 77030, USA; sprasad@mdanderson.org (S.P.); amittyagiiitd@gmail.com (A.K.T.); lokeshdeb.ibsd@nic.in (L.D.); zshuangjiamin@126.com (J.H.); bbaggarwa@gmail.com (B.B.A.)

**Keywords:** apoptosis, survival proteins, chemotherapeutics, nutraceuticals, Bcl-2 family, surviving

## Abstract

Escaping from cell death is one of the adaptations that enable cancer cells to stave off anticancer therapies. The key players in avoiding apoptosis are collectively known as survival proteins. Survival proteins comprise the Bcl-2, inhibitor of apoptosis (IAP), and heat shock protein (HSP) families. The aberrant expression of these proteins is associated with a range of biological activities that promote cancer cell survival, proliferation, and resistance to therapy. Several therapeutic strategies that target survival proteins are based on mimicking BH3 domains or the IAP-binding motif or competing with ATP for the Hsp90 ATP-binding pocket. Alternative strategies, including use of nutraceuticals, transcriptional repression, and antisense oligonucleotides, provide options to target survival proteins. This review focuses on the role of survival proteins in chemoresistance and current therapeutic strategies in preclinical or clinical trials that target survival protein signaling pathways. Recent approaches to target survival proteins-including nutraceuticals, small-molecule inhibitors, peptides, and Bcl-2-specific mimetic are explored. Therapeutic inventions targeting survival proteins are promising strategies to inhibit cancer cell survival and chemoresistance. However, complete eradication of resistance is a distant dream. For a successful clinical outcome, pretreatment with novel survival protein inhibitors alone or in combination with conventional therapies holds great promise.

## 1. Introduction

**Figure 1:**
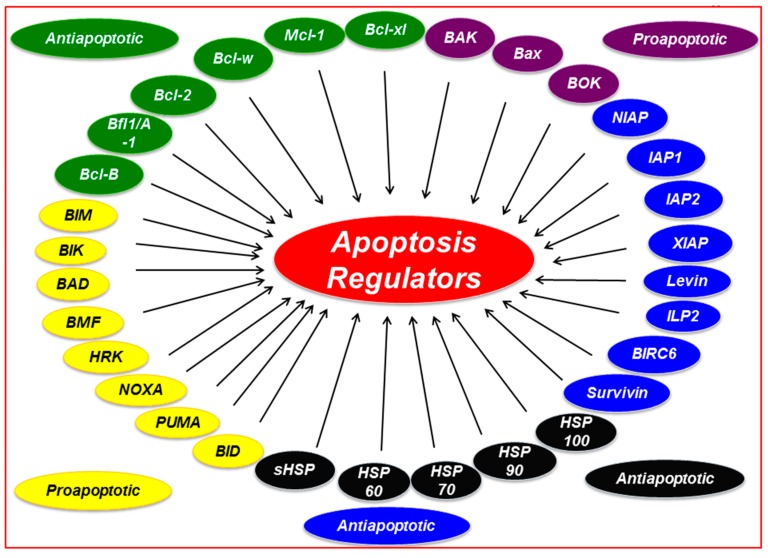
Survival family members associated with regulation of apoptosis. Abbreviations: Mcl-1, myeloid cell leukemia 1; Bcl-xL, B-cell lymphoma-extra-large; Bcl-2, B-cell CLL/lymphoma 2; Bcl-w, Bcl-2 like 2; BIM, Bcl-2 interacting protein; BIK, Bcl-2 interacting killer; BAD, Bcl-2 antagonist of cell death; BID, BH3 interacting domain death agonist; NOXA, Phorbol-12-myristate-13-acetate-induced protein 1; PUMA, p53 upregulated modulator of apoptosis; HRK, harakiri; BOK, Bcl-2 related ovarian killer; BAK, Bcl-2 antagonist killer1; BAX, Bcl-2 associated X protein; IAPs, Inhibitors of apoptosis; HSP, heat-shock protein.

Homeostasis in multicellular organisms is tightly maintained by a process known as programmed cell death or apoptosis. The aberrant regulation of apoptosis is associated with tumorigenesis and, importantly, in the development of chemoresistance. Several proteins that have been linked with dysregulated programmed cell death are collectively called survival proteins. Bcl-2, inhibitor of apoptosis (IAP), and heat shock protein (HSP) are considered to be the main survival protein families (Figure 1). The survival proteins impede cell death thus provide targets for possible drug discovery and development. This review describes the role of survival proteins in chemoresistance and summarizes some of the more promising strategies/agents for the modulation of survival proteins in cancer therapy.

### 1.1. Bcl-2 Family

In the past several decades, Bcl-2 family proteins have gained attention as apoptosis regulators. The Bcl-2 family consists of proteins that determine the fate of cells by promoting or inhibiting apoptosis. First identified in 1985 by Tsujimoto *et al.*, to date, more than 25 Bcl-2 proteins have been characterized [1]. All Bcl-2 proteins are characterized by the presence of up to four Bcl-2 homology (BH) domains; these proteins can be divided into three subfamilies based on their structure and functions [2]. Group 1, the anti-apoptotic subfamily, contains the Bcl-2, Bcl-xL, Bcl-w, Mcl-1, Bfl1/A-1, and Bcl-B proteins, which suppress apoptosis and may contain all four BH domains. Group 2, a pro-apoptotic subfamily, contains the BAK, BAX, and BOK proteins, which are localized to the mitochondria, smooth endoplasmic reticulum, and perinuclear membranes and contain BH 1–3 domains. Group 3 consists of the pro-apoptotic BIM, BID, BIK, BAD, BMF, HRK, PUMA, and NOXA proteins, which contain only the BH3 domain and are known as “BH3-only” proteins [3,4].

In cancer cells, resistance to therapy evolves by various mechanisms. Among the most important of these mechanisms are the overexpression of anti-apoptotic genes and the downregulation or mutation of pro-apoptotic genes [5]. Extensive studies have established the role of the Bcl-2 family of pro-survival proteins in cell survival, proliferation, and chemoresistance in various tumors [6,7]. Based on their adverse role in patient outcomes, much attention has been given to this family in the search for new therapeutic regimens.

#### 1.1.1. Bcl-2

The overexpression of the Bcl-2 protein is associated with malignancy and with poor prognosis in various cancers [8,9,10,11,12,13,14]. Several studies have shown that higher expression of Bcl-2 leads to resistance against radiation and chemotherapy [12,15]. How Bcl-2 is overexpressed in cancer cells is not fully understood; however, Bcl-2 overexpression is correlated with chromosomal deletions leading to loss of Bcl-2—targeting miRNAs such as miR-195, miR-24-2, and miR-365-2 [16,17]; gene amplification [18]; and hypermethylation of the *BCL2* gene [19]. Furthermore, several studies in the 1990s showed that the overexpression of pro-survival Bcl-2 proteins alone is not tumorigenic but that the concurrence of additional mutation(s), along with the overexpression of these proteins, is associated with chemoresistance in several malignancies [20,21,22,23,24].

#### 1.1.2. Bcl-xL

Bcl-xL, an anti-apoptotic member of Bcl-2 family, also has been investigated for its role in treatment resistance [6,25]. The overexpression of Bcl-xL is associated with poor prognosis and chemoresistance in both hematological malignancies and solid tumors [7,26,27].

#### 1.1.3. Mcl-1

Mcl-1, which was first identified in human myeloid leukemia cell line ML-1, structurally resembles other anti-apoptotic Bcl-2 proteins [28]. However, Mcl-1 differs from other anti-apoptotic Bcl-2 proteins in its BH3 domain binding pattern, which involves the BH3 domain [29]. Several studies have suggested that Mcl-1 is required for embryonic development [30], survival of hematopoietic stem cells [31], and the survival of multiple cell lineages, including lymphocytes [32] and neurons [33]. As with other Bcl-2 family members, the overexpression of Mcl-1 has been reported in several malignancies [34,35], and its expression is often associated with treatment resistance, relapse [36], and poor survival outcomes [37,38]. In normal cells, the expression of Mcl-1 is regulated by both ubiquitin-dependent and ubiquitin-independent degradation [34,39,40]. To date, three E3 ubiquitin ligases have been identified that are involved in the regulation of Mcl-1 expression [40,41,42].

#### 1.1.4. Pro-Apoptotic Proteins

As discussed above, chemoresistance not only may result from the overexpression of anti-apoptotic proteins but also from the inactivation of pro-apoptotic proteins. Along these lines, the inactivation of BAX is correlated with several malignancies [43,44]. Similarly, the inactivation of NOXA and PUMA are linked with tumorigenesis and treatment resistance [45,46].

### 1.2. IAP Family

The IAP family proteins are another essential component in the survival of cancer cells. The overexpression of IAP proteins is associated with cancer cell survival, treatment resistance, and poor prognosis. Members of the IAP protein family were first discovered in the genetic screening of baculoviruses *Orgyia pseudotsugata* and *Cydia pomonella* [47,48,49]. Since then, eight IAP proteins have been identified in humans: NIAP, also called neuronal apoptosis inhibitory protein or BIRC1; c-IAP1, also called cellular IAP1 or BIRC2; c-IAP2, also called cellular IAP2 or BIRC3; XIAP, also called X chromosome–linked IAP or BIRC4; survivin, also called BIRC5; BIRC6, also called ubiquitin-conjugating BIR domain enzyme apollon; livin, also called melanoma IAP, ML-IAP, or BIRC7; and ILP2, also called IAP-like protein 2 or BIRC8 [47,50].

The IAP family members control apoptosis through several mechanisms. The best understood of these mechanisms is caspase inhibition; others include the regulation of cell division, cell cycle progression, and signaling pathways. For example, XIAP and NAIP form a complex with the TAK1 kinase and its cofactor, TAB1, which leads to activation of c-Jun-NH_2_-terminal kinase 1 [51]. Furthermore, XIAP facilitates NF-κB activation, which contributes to tumorigenesis [52,53].

All IAP proteins contain the unique baculoviral IAP repeat (BIR) domain. Some IAP proteins (c-IAP1, c-IAP2, XIAP, and livin) also contain a carboxy-terminal RING domain. IAP proteins containing a RING domain work as E3 ubiquitin ligases and play important roles in cell survival and signaling pathways [54,55]. Based on the presence of the BIR and RING domains, this family is subdivided into three classes [56].

#### 1.2.1. Class 1 IAPs

XIAP, cIAP1, cIAP2, ILP-2, and livin constitute the class 1 IAPs. These proteins contain homologous BIR domains and a RING finger motif. XIAP was first discovered, and among the members it is the best characterized [57]. It has three BIR and one RING finger domain. It has been shown that XIAP inhibits caspases 3, 7, and 9, but not caspase 8 [58,59]. cIAP1 and cIAP2 are structurally homologous with XIAP, with three BIR and one RING finger domain; however, they have a weaker binding affinity to caspase than does XIAP [59]. Livin and ILP-2 contain a RING finger and only one BIR domain [56]. Livin is highly expressed in melanoma and inhibits caspases 3 and 9 but not caspases 1, 2, 6, or 8 [60]. ILP-2 inhibits caspase 9, but not caspases 3, 7, or 8 [61].

#### 1.2.2. Class 2 IAPs

The only member of class 2, NIAP, has three BIR domains but no RING finger motif [62]. It inhibits caspases 3 and 7, but not caspases 1, 4, 5, or 8 [63].

#### 1.2.3. Class 3 IAPs

The very important and well-studied member of class 3 is survivin. Survivin contains a single BIR domain and no RING finger. Survivin is overexpressed in a variety of malignancies [64,65,66,67,68]. Since survivin is expressed in normal cells, the differential expression between normal and malignant cells can be exploited for therapeutic purposes.

### 1.3. HSP Family

HSPs are a ubiquitous family of molecular chaperones, classified into subfamilies according to their molecular size. The HSP subfamilies are HSP100, Hsp90, Hsp70, Hsp60, and small HSPs (size varying from 15 to 30 kDa) [69,70]. With adaptor molecules and co-chaperones, HSPs form a large multiprotein complex and regulate more than 200 proteins [71,72,73].

One of the most studied and characterized members of the HSP family is Hsp90. Hsp90 is highly conserved from unicellular organisms to mammals. High expression of Hsp90 is associated with many solid tumors and hematological malignancies. The overexpression Hsp90 has been linked with cancer cell survival and proliferation [74,75]. In addition, Hsp90 is required for the maturation and functional stability of various proteins essential for cancer cell immortality, survival, anti-apoptosis, metabolism, genomic instability, and metastasis [76]. Since its interacting partners are oncogenes, mutated genes, or proteins that are overexpressed in tumor cells and involved with tumorigenesis, Hsp90 represents a promising therapeutic target [71,77,78].

## 2. Inhibitors of Survival Proteins

In the past several decades, the survival protein families have been targeted in cancer treatment strategies. Several types of survival protein–inhibiting agents have been developed, including antisense oligonucleotides (ASOs); peptides, small-molecule inhibitors, and nutraceuticals. Some such agents are under investigation in clinical trials, alone or combined with other therapies (Table 1).

### 2.1. Inhibitors of Bcl-2 Proteins

Multiple strategies have been adopted to target the Bcl-2 family members, including the use of peptides, small molecules, and ASOs.

#### 2.1.1. Peptide-Based Inhibitors

Sattler *et al.* provided first evidence that a BAX-BH3 peptide could inhibit the anti-apoptotic activity of Bcl-xL by binding to its hydrophobic groove [79]. Later studies of the crystal structure of all BH3-only proteins revealed that each BH3-only protein binds specifically with another anti-apoptotic protein. For example, the BH3-only protein NOXA can only bind with Mcl-1 [80]. This understanding of the intimate relationship of BH3-only proteins with anti-apoptotic proteins led to the discovery of several BH3 mimetic peptides. For example, a peptide derived from the nuclear receptor Nur77 was shown to bind between the BH3 and BH4 domains, unmasking the BH3 domain and leading to a functional switch of Bcl-2 from an anti-apoptotic to a pro-apoptotic protein [81]. Another strategy popularly known as “stapled peptides” was developed to target the BH3 domain of BIM, inhibiting Bcl-2-BIM interactions. This stapled peptide successfully overcomes apoptotic resistance in hematologic cancers [82]. These proof-of-principle studies are encouraging; however, a cautious approach and more studies are required before BH3 peptides can be widely adopted as therapeutic agents.

#### 2.1.2. ASOs

First used against Bcl-2, ASOs are another tool to inhibit the expression of anti-apoptotic Bcl-2 family proteins [83,84]. ASOs are synthetic, short, single-stranded DNA molecules that can interfere with gene expression by forming a heteroduplex with complementary sequences within target messenger RNAs. Nonetheless, several attempts have been made successfully in this regard to develop specific ASO inhibitors against Bcl-2 family, and few of them are now in clinical trials. Phase II trials of oblimersen, also called G-3139, for the treatment of leukemia, lymphoma, and cancers of the prostate, colon, and breast are under way, as are phase III trials of its use against melanoma and myeloma [5]. However, oblimersen failed to get FDA approval for the treatment of relapsed or refractory chronic lymphocytic leukemia, since it did not show significant survival improvements compared with the standard of care for such in patients [85]. Combinations of ASOs with existing chemotherapeutic agents have shown the promise against several cancers [86,87,88]. Despite the encouraging results from recent studies, the use of ASOs is limited because of their non-specific binding and lack of stability [89].

#### 2.1.3. Small-Molecule Inhibitors

**Figure 2:**
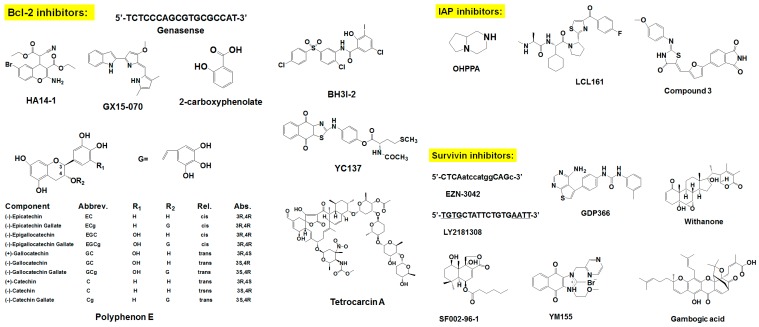

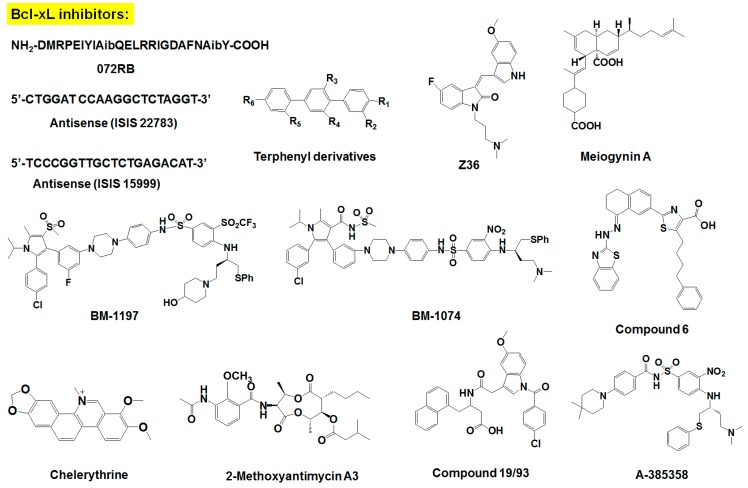

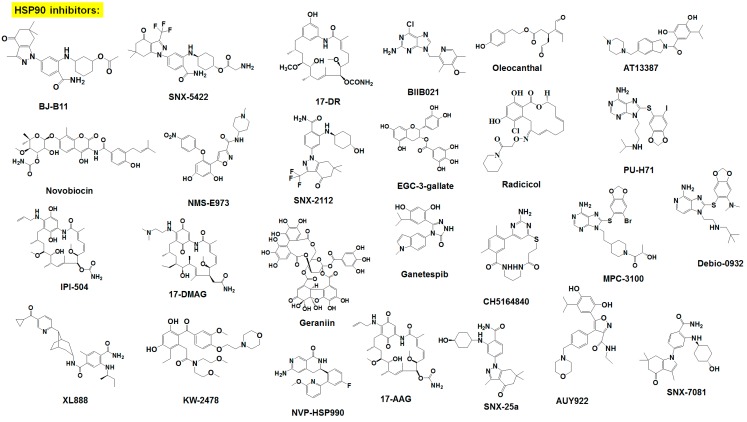

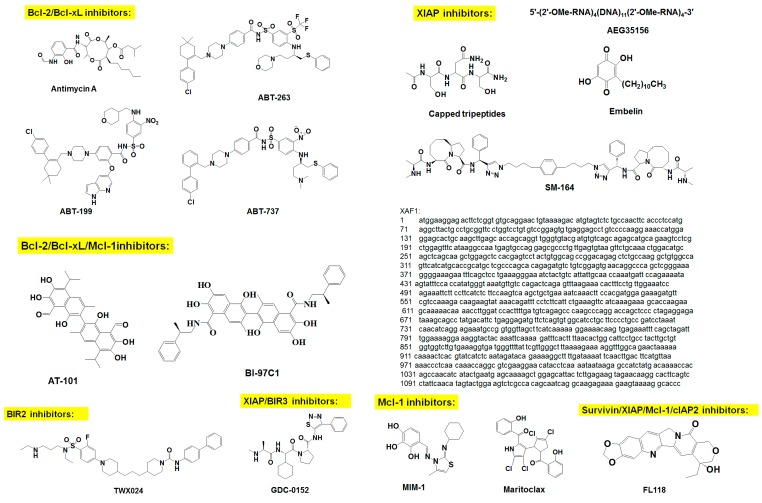
Chemical structure of survival family protein inhibitors.

Organic compounds smaller than 900 Da are considered small-molecule compounds. Because of their size, these smaller agents have greater potential than ASOs and peptides as Bcl-2 inhibitors. Several screening strategies have been adopted to find specific small-molecule inhibitors of anti-apoptotic Bcl-2 family members. Most small-molecule inhibitors have been developed as BH3 mimetics, since BH3 binding to the cleft of Bcl-2 are critical for Bcl-2’s anti-apoptotic activities. Small-molecule inhibitors derived from natural products also have shown promising efficacy in variety of cancers [90,91,92,93]. ABT-737, navitoclax, obatoclax, maritoclax, gossypol and its derivatives, and BH3-M6 are some of the small-molecule inhibitors currently in preclinical and clinical phases of development (please see Table 1 and Figure 2).

To date, the BH3 mimetic ABT-737 and its orally active analogue navitoclax (also called ABT-263) are the most potent Bcl-2 and Bcl-xL inhibitors. As a single agent, ABT-737 is effective in several preclinical models and sensitizes tumors to radiation and chemotherapy [94,95,96,97]. Importantly, phase I/II clinical trials of navitoclax against several cancers have been encouraging [98,99,100]. However, recent studies suggest that cancer cells develop resistance to ABT-737 through the upregulation of Mcl-1 [101], and in many instances this resistance can be overcome by the downregulation of Mcl-1 [90,91,102,103]. Thus, the simultaneous targeting of Bcl-2 and Mcl-1 could improve patient outcomes.

Obatoclax, also called GX015-070, is a pan–Bcl-2 inhibitor derived from prodiginines. Obatoclax inhibits the expression of Mcl-1 and other members of the Bcl-2 family. Studies have suggested that Obatoclax is effective against several cancers as single agent or combined with other agents [104,105,106]. Phase III clinical trials of obatoclax are ongoing; however, neurotoxicity caused by this agent may obstruct further progress [100,107,108].

Recently another Mcl-1 inhibitor, maritoclax (also called marinopyrrole A), was identified from marine species of streptomycetes [109]. Doi *et al.* suggested that maritoclax inhibits Mcl-1 via direct binding and proteasomal degradation [90]. Maritoclax was found to be effective in Mcl-1 overexpressing cells [90,91,110]. The discovery of maritoclax was exciting because the molecule binds specifically with Mcl-1 and increases the efficacy of ABT-737 in various cancer cells lines [91,110]; however, further pharmacokinetic studies are needed to translate this agent to clinic.

Gossypol was first identified as a male contraceptive in cottonseeds and is now being developed as an anticancer agent. Several studies have shown that gossypol and its derivatives bind to the hydrophobic grooves of Bcl-2 and Bcl-xL [111]; however, gossypol is not effective against Mcl-1. Gossypol is presently being investigated in clinical trials [112].

The second-generation benzenesulfonyl derivative of gossypol, TW-37, is a promising agent [113] that inhibits Bcl-2, Bcl-xL, and Mcl-1. Studies suggest that TW-37 is effective as single agent and potentiates the apoptotic effects of chemotherapeutic agents [114,115].

The third-generation of gossypol derivative, apogossypolone (also called ApoG2), also has been shown to inhibit Mcl-1 and other members of the Bcl-2 family. Preclinical studies suggest that apogossypolone is an effective agent against hematological malignancies [116].

A (−) enantiomer of gossypol, AT-101, has shown great promise against lymphoma. AT-101, currently studied in phase II clinical trials, is effective as single agent and potentiates the cytotoxic effects of several therapeutic agents [6,117].

Sabutoclax, also called BI-97C1, is the newest derivative of gossypol. Sabutoclax binds with Bcl-2, Mcl-1, and Bfl-1 with a better affinity than do other gossypol derivatives. In preclinical studies, sabutoclax was shown to induce apoptosis in ABT-737-resistant diffuse large B-cell lymphoma cells and to be effective in various cancer cell lines and xenograft models [118,119].

Designed as a pan–Bcl-2 antagonist, BH3-M6 is the most recent BH3 mimetic. BH3-M6 inhibits proteins in the anti-apoptotic subfamily [120]. Further pharmacokinetic studies are required to fully evaluate this agent.

### 2.2. Inhibitors of IAP Family Proteins

Because of their constitutive expression in a variety of tumors, IAP family members have been attractive therapeutic targets. Among the strategies that have been explored to target IAP family members, the development of small-molecule antagonists has been the most studied. IAP antagonists either directly bind to IAP family proteins to prevent the proteins’ binding to caspases and SMAC or stimulate ubiquitination and proteasomal degradation by inducing conformation changes [121,122]. The following IAPs antagonists have been or are currently being tested in preclinical or clinical studies.

#### 2.2.1. Selective IAP Inhibitors

Because IAP family proteins differ in their structure and function, strategies have been adopted to selectively target individual members of IAP family. CS3 has been developed as selective antagonist of c-IAP1 and c-IAP2 [47,123]. Studies have suggested that CS3 degrades c-IAP1 and c-IAP2, but also activates the NF-κB signaling pathway; its therapeutic potential is therefore limited [123]. Another pan-selective IAP antagonist, PS1, has been shown to be a more potent antagonist than CS3 [123]. Recently, high-throughput screening identified TWX-024 as an XIAP-selective antagonist that disrupts XIAP-caspase 3 interaction [124].

#### 2.2.2. Inhibitors Mimicking SMAC

The discovery of bivalent SMAC mimetic agents led to their development as a new class of IAP inhibitors [125]. These bivalent SMAC mimetics are made up of two monovalent units linked by a chemical structure [126] and have been shown to promote the dimerization of the BIR2-BIR3 domains of c-IAP1, as well as BIR3 constructs of XIAP [127]. Several studies have suggested that these SMAC mimetics inhibit cell proliferation and tumor growth [128].

#### 2.2.3. SMAC-Derived Peptides

The characterization of SMAC and BIR domains led to the understanding of the precise region involved in the binding of peptides to selected BIR domains [129,130,131]. Once this mystery was solved, the synthesis of SMAC peptides became reality. Along these lines, peptides derived from the N terminus of mature active SMAC were found to mimic the activity of the SMAC protein and thus inhibit IAP proteins [132]. Further studies revealed that SMAC peptides blocked IAP-caspase interaction and sensitizes effects of TRAIL in the xenograft model [133,134]. Further studies and refinements of the pharmacological properties of SMAC peptides will be required before their use for the treatment of human cancers.

### 2.3. Inhibitors of Hsp90

The understanding of Hsp90 function and its association with cancer steered therapeutic scientists toward the development of Hsp90 inhibitors as a potential strategy for cancer treatment. A few Hsp90 inhibitors are currently being tested in the preclinical models, and some are under clinical investigation.

#### 2.3.1. Derivatives of Geldanamycin

Geldanamycin, a benzoquinone ansamycin antibiotic, inhibits Hsp90 via binding to the ADP/ATP-binding pocket of the protein [71]. Because of its unfavorable pharmacological properties, which include hepatotoxicity and poor solubility, several attempts have been made to modify geldanamycin. By substituting a methoxy group for an allyl amino group, a geldanamycin derivative, tanespimycin (17-allylamino-17-demethoxy-geldanamycin; 17-AAG), was developed [135]. Studies have shown that tanespimycin, alone or in combination with chemotherapeutic drugs, is effective against a variety of cancers [71,136]. The manufacturer is engaged in further modification of this agent [137,138].

#### 2.3.2. Resorcinol and Its Derivatives

Resorcinol, a natural antibiotic that competes with ATP for Hsp90 binding, is a potent inhibitor of Hsp90. However, *in vivo* studies of resorcinol’s efficacy were not encouraging; therefore, several attempts have been made to modify the antibiotic. Along those lines, several resorcinol derivatives have been developed, such as NVP-AUY922, AT-13387, STA-9090, and KW-2478 [139]. Studies suggest that these derivatives are more potent than resorcinol [140].

#### 2.3.3. Purine-Based Inhibitors

Chiosis *et al.* revealed another potential Hsp90 inhibitor, PU-H71 [141]. PU-H71, a synthetic molecule that competes with ATP for the Hsp90 ATP binding pocket, was found to be efficacious against breast cancer [142]. BIIB021, CUDC-305, and NVP-BEP800 are derivatives of PU-H71 and have found to be efficacious against several cancers [143,144]. BIIB021 is now in clinical development [145].

### 2.4. Natural Agents as Survival Protein Inhibitors

For several decades it has been of great interest to identify and characterize potential anti-cancer agents derived from natural resources, particularly those derived from spices, fruits, and vegetables. Many spice-derived nutraceuticals have been shown to help induce apoptosis and reduce inflammation and levels of survival proteins. Extensive work from our group and others has found that ajoene, butein, curcumin, dihydroartemisinin, diosgenin, ellagic acid, embelin, emodin, epigallocatechin gallate, escin, eugenol, garcinol, genistein, geraniin, guggulsterone, resveratrol, rosmarinic acid, and zerumbone have great potential to inhibit various survival proteins.

Because these agents possess high antioxidant and anti-inflammatory properties and target multiple pathways, these agents are considered “magic bullets” [146]. For this reason, many researchers are trying to develop new drugs derived from natural sources. Some such natural-derived agents are already in clinical trials; these include curcumin and gossypol [147,148].

Curcumin is the chief polyphenol in turmeric and is known to bring about apoptosis in cancer cells. Curcumin upregulates the pro-apoptotic proteins BIM, BAK, BAX, NOXA, and PUMA and downregulates the anti-apoptotic proteins Bcl-2 and Bcl-xL [146]. An *in vitro* study in human multiple myeloma cells demonstrated that curcumin downregulated survivin and Bcl-2 expression and upregulated BAX expression, leading to apoptosis [149]. Rosmarinic acid was shown to induce apoptosis and downregulate Bcl-2 in an *in vitro* study of human T-cell leukemia [150]. Several spice-derived nutraceuticals target caspases, which are necessary for apoptosis. Some natural compounds stimulate caspase activity while suppressing Bcl-2 or Bcl-xL expression. For example, in an *in vitro* study of human myelogenous leukemia cells, ajoene was shown to activate caspase-3 and cleave Bcl-2 [151].

## 3. Conclusions and Perspectives

The large volume of studies in various cancers over recent decades has provided unequivocal evidence that the overexpression of survival proteins is associated with cancer cell proliferation, survival, and chemoresistance. However, further studies are required to understand the regulation of the survival proteins, their tissue dynamics, and resistance mechanisms. There is a persistent need to develop novel strategies and agents to combat the resistance to chemotherapy and radiation observed in cancers. The ultimate goal would be to develop a novel agent that could expel the surviving cancer cells that are primarily resistant to conventional therapies.

A major weakness of current survival protein-targeting therapy strategies is their inability to target all survival proteins with the same affinity. Another problem is the unfavorable pharmacological properties of some promising agents. Thus, it is very important to understand the role of each individual survival protein family. For example, most inhibitors of pro-survival Bcl-2 proteins are weak Mcl-1 inhibitors; therefore, treating with those agents may not provide any therapeutic advantage for cancers whose main driver is Mcl-1. Thus, targeting Bcl-2 along with Mcl-1 should be an attractive option; however, a very careful approach is needed to consider this strategy. High-throughput screening technologies coupled with bioinformatic approaches will continue to be employed in the coming years to develop novel drugs against crucial players in cancer development and progression. In our opinion, the future development of more targeted inhibitors that have higher and broader affinities for all the major survival proteins, as well as agents that regulate cancer-signaling pathways, should greatly improve patient survival and prevent tumor recurrence or resistance.

## Figures and Tables

**Table 1:** A list of inhibitors of cell survival proteins identified as potential therapeutics at different stages of clinical development.

Compound	Reference	Compound	Reference
***Bcl-2 inhibitor***		***Survivin inhibitor***	
GX15-070 **	[152]	LY2181308 **	[153]
HA-14	[154]	Ad-Survivin T34A	[155]
HA14-1	[156]	EZN-3042 *	[157]
BH3I-1/BH3I-2	[158]	SPC3042	[159]
2-carboxyphenolate	[160]	YM155 **	[161]
Genasense ***	[85]	SF002-96-1	[162]
Polyphenon E **	[163]	Withanone	[164]
SAHBs	[165]	GDP366	[166]
YC137	[167]	Gambogic acid	[113]
Tetrocarcin-A derivatives	[168]	***Mcl-1 inhibitor***	
***Bcl-xL inhibitor***		Maritoclax	[90]
Chelerythrine	[169]	MIM-1	[170]
Compound 6	[171]	***BIR2 inhibitor***	
2-Methoxyantimycin A3	[172]	TWX024	[173]
BM-1197	[174]	***Survivin/XIAP/Mcl-1/cIAP2 inhibitor***	
BM-1074	[175]	FL118	[176]
Compound 19/93	[177]	***HSP90 inhibitor***	
Z36	[178]	SNX-25a	[179]
072RB	[180]	Novobiocin	[181]
A-385358	[182]	17-DR	[183]
Antisense (ISIS 15999)	[184]	Debio 0932	[185]
Antisense (ISIS 22783)	[186]	SNX-2112	[187]
Terphenyl derivatives	[188]	PU-H71	[142]
Meiogynin A	[189]	AT13387	[190]
***BCl-2/Bcl-xL inhibitor***		NMS-E973	[191]
Nativoclax (ABT-263) **	[192]	NXD30001	[193]
ABT-199 **	[194]	Geraniin	[195]
Antimycin A	[92]	CH5164840	[196]
BM-957	[197]	EGC-3-gallate	[198]
ABT-737	[199]	Oleocanthal	[200]
***Bcl-2/Bcl-xL/Mcl-1 inhibitor***		XL888	[201]
Gossypol (AT-101) **	[148]	SNX-7081	[202]
BI-97C1	[118]	NVP-HSP990	[203]
***XIAP inhibitor***		Radicicol	[204]
BIR3 antagonists	[205,206]	BJ-B11	[207]
PPU derivatives	[208]	KW-2478	[209]
Capped tripeptides ^205^		MPC-3100	[210]
SM-164	[127]	Peptide PEP73	[195]
AEG35156 **	[211]	AUY922 *	[212]
Embelin	[213]	17-DMAG *	[214]
***XIAP/BIR3 inhibitor***		SNX5422 *	[215]
GDC-0152	[216]	BIIB021 **	[217]
***IAIAP inhibitor***		17-AAG **	[218]
LCL161	[219]	Ganetespib **	[220]
OHPPA	[221]	IPI-504 **	[222]
Livin (ML-IAP)	[223]		

* Phase I; ** Phase II; *** Phase III; Compound 6, (*E*)-2-(8-(2-(Benzo[d]thiazol-2-yl)hydrazono)-5,6,7,8-tetrahydronaphthalen-2-yl)-5-(4-phenylbutyl)thiazole-4-carboxylic acid; Compound 19/93, (*R*)-3-(amido indomethacin)-4-(naphthalen-1-yl)butanoic acid; SAHBs, stabilized alpha-helices of BCL-2 domains; 17-AAG, 17-allylamino, 17-demethoxygeldanamycin; 17-DR, 17-Demethoxy-reblastatin; EGC, Epigallocatechin; PPU, Polyphenylurea; OHPPA, octahydropyrrolo[1,2-a]pyrazine A.
